# The Complex Advertisement Call Characteristics of *Pelophylax terentievi* May Convey Physical Information

**DOI:** 10.3390/biology15151304

**Published:** 2026-08-05

**Authors:** Yan Wang, Zihang Xu, Yaming Sun, Mengmeng Gong, Abida Iminjan, Azhar Orkax, Xuyang Ma, Jingwen Wang, Lu Zhou

**Affiliations:** Xinjiang Key Laboratory for Ecological Adaptation and Evolution of Extreme Environment Organisms, College of Life Sciences, Xinjiang Agricultural University, Ürümqi 830052, China; 17881153852@163.com (Y.W.); 19253962938@163.com (Z.X.); 18335239112@163.com (Y.S.); 19710215329@163.com (M.G.); 15276173075@163.com (A.I.); 18160535314@163.com (A.O.); 13895026363@163.com (X.M.); 18605353118@163.com (J.W.)

**Keywords:** Central Asian pond frog, advertisement call, acoustic parameters, diel rhythms, body size relationship

## Abstract

Advertisement calls convey individual information and play a vital role in amphibian reproduction. Body size is one of the key factors determining the diversity of frog advertisement calls. *P. terentievi* is distributed in mountainous areas across Tajikistan and Xinjiang, China. However, bioacoustic research on this species remains an unexplored field. In this study, the advertisement calls of 20 male frogs were recorded in the breeding season and six distinct call types were described based on spectrum morphology and auditory characteristics. The coefficient of variation analysis indicates some of their call types may serve as effective cues for individual recognition. Their advertisement calls exhibit a typical sentence structure and often conclude with a distinctive ending word. We also observed a distinct diel rhythm in their calls: advertisement calls begin before sunset, peak after sunset, and continue until noon. Furthermore, their acoustic parameters are significantly correlated with their head size, leg size and body weight. Males with larger heads and longer legs often make calls at higher frequencies, possibly to stand out in the louder chorus to enhance the efficiency of attracting females. This basic study can provide support for the behavioral and evolutionary biology research of *Pelophylax* species.

## Simple Summary

*Pelophylax terentievi* is a typical mountainous frog across Tajikistan and Xinjiang, whose vocalizations have not been revealed yet. Six advertisement call types were identified, most of which can serve as effective cues for individual recognition. Moreover, its advertisement calls exhibit a typical sentence structure and often conclude with a distinctive ending word. Its calling diel rhythm is negatively correlated with air temperature. Males with larger heads and longer legs often make calls at higher frequencies, possibly to stand out in the louder chorus to enhance the efficiency of attracting females.

## 1. Introduction

The vocalizations produced by male anurans serve crucial functions in species recognition, female sexual selection, and homosexual competition [1,2,3,4]. Most amphibians initiate reproductive activities with advertisement calls [5,6,7]. Male frogs use advertisement calls to establish territorial dominance, engage in display behaviors, and participate in other competitive behaviors [8,9], and these calls attract females to breeding sites for mating [10,11,12] and stimulate ovulation [13]. The biological relevance of amphibian vocalizations, coupled with their relative simplicity, makes them a quintessential trait for studying sexual selection and speciation [14,15,16].

The vocalizations of anurans exhibit varying levels of interspecific and intraspecific variation [17,18]. There is a growing recognition within the scientific community that both types of variation can significantly influence population, community, and ecosystem dynamics [19]. The variation in the frequency of calls among individuals within a population is often influenced by body size or genetic differences [17,20], while the variation in call duration may be affected by temperature. The differences in call frequency between different populations of the same species may be influenced by temperature and humidity, geographical isolation, and genetic differentiation [21,22]. The advertisement calls of different species within the same genus are usually similar, such as *P. bedriagae* and *P. caralitanus*, both consisting of multi-syllabic pulses [23,24]. However, sometimes there can be significant differences, such as *P. ridibundus* and *P. nigromaculatus*, which are composed of multi-syllabic harmonics [25,26,27,28]. The differences in calls between species may be influenced by genetics and environmental differences in their distribution areas [29,30]. Traits with a coefficient of variation (CV) below 5% are commonly designated as “static” traits, whereas traits with a CV above 12% are referred to as “dynamic” traits. These dynamic traits have been observed to demonstrate substantial inter-individual variation, making them useful markers for individual recognition [17]. Numerous studies on *Uperodon systoma* and *U. globulosus* have consistently shown that the coefficient of variation between individuals (CV_a_) exceeds the coefficient of variation within individuals (CV_w_) [31].

The diel rhythms of frog calls play a crucial role in ensuring communication efficiency [32,33]. Currently, research on the rhythms of frog vocalizations has primarily focused on species that exhibit nocturnal calling behavior. In contrast, studies on species that exhibit diurnal calling behavior are relatively scarce [34,35]. Frogs demonstrate the capacity to dynamically adjust call parameters or increase the number of calls in response to environmental pressures (such as the number of competitors or predation risk). Through these adjustments, they strike a balance between communication efficiency, energy expenditure, and survival risk [36]. Investigating the calling diel rhythms of specific species is instrumental in comprehending vocal communication within a group and plays a pivotal role in their reproductive biology.

Many studies have documented substantial variations among frogs with respect to body size, tympanic membrane characteristics, and vocal frequency. These species also exhibit rich diversity in their reproductive behaviors [37,38]. Body size has been demonstrated to influence the vocal characteristics of anurans. Generally, larger individuals (defined as such in terms of weight or body length) exhibit lower vocal frequency, longer vocalization duration, and higher calling rate. These characteristics give them an advantage in male competition and female selection [39]. For instance, in frogs, body size has been shown to exhibit a negative correlation with the peak frequency of their calls [40,41]. The peak frequency of *Rhacophorus dennysi* has a significant negative correlation with body weight [42]. Analogous results were obtained in studies of the midwife toad (*Alytes obstetricans* and *A. cisternasii*) and the European tree frog (*Hyla arborea*) [43,44].

*P. terentievi* is distributed in the mountainous regions of Tajikistan and Xinjiang, China. In Xinjiang, the species is primarily found in still-water habitats beside marshes, riverbanks, farmlands, and grasslands, at elevations ranging from 500 to 2000 m [45] (pp. 1071–1075). For a long time, this species was classified as *Rana ridibunda*. However, subsequent morphological and molecular biological studies have since determined it to belong to the genus *Pelophylax* in the family Ranidae [46]. The vocal characteristics of *P. terentievi* may directly influence its reproductive strategies and population persistence. However, previous studies on this species primarily focused on taxonomy and morphology and seldom involved behaviors, constraining our understanding of the species’ ecology and evolutionary biology.

This study focuses on the advertisement calls of *P. terentievi* and takes a systematic approach to investigate the vocal characteristics, vocal rhythms, and relationship between vocalizations and body size during its breeding season. The findings will supplement fundamental data on the acoustics of *P. terentievi* and provide support for the reproductive adaptation strategies of anura in arid and semi-arid regions.

## 2. Materials and Methods

### 2.1. Data Collection

The selection of sampling sites was made from rural ponds located to the south of Shihezi City, situated on the northern slope of the Tianshan Mountains. In May 2025 (breeding season), after sunset, advertisement calls were recorded using a Sony A10 recorder (Sony Co., Ltd., Tokyo, Japan) with a directional microphone at a sampling rate of 44.1 kHz. The microphone was positioned at a distance of approximately 1 m from a calling individual [47]. The advertisement calls of the 20 male frogs were recorded individually. Each male was recorded for approximately 30 min. Subsequent to the recording, a series of morphological measurements were obtained, including snout–vent length (mm), head length (mm), head width (mm), snout length (mm), internarial distance (mm), interorbital distance (mm), width of upper eyelid (mm), eye diameter (mm), tympanum diameter (mm), forearm and hand length (mm), forearm width (mm), total length of the hindlimb (mm), tibia length (mm), foot length (mm), and body weight (g) [46]. Furthermore, an omnidirectional microphone recorder (Song Meter Mini, Wildlife Acoustics Co., Ltd., Maynard, MA, USA, sampling rate of 44.1 kHz) was positioned at the edge of another pond in order to conduct continuous 24 h recordings of the entire pond for the purpose of call rhythm data collection (the recording here lasted for one day). Concurrently, the air temperature (°C), water temperature (°C) and humidity (%) were measured. Upon completion of the morphological measurements, the animals were released back to their original capture sites.

### 2.2. Acoustics

The audio files were subjected to Fast Fourier Transform analysis using Raven Pro 1.6, thereby generating waveforms and spectrograms. The classification process was conducted manually based on the auditory and visual characteristics of *P. terentievi* advertisement calls [48]. The classification criteria were as follows: audibly based on the timbre of the sound, the duration and roughness of the sound; visually based on the type of the signal, the shape of the harmonics, the number of notes, the number of harmonics or pulses, the number of inflection points, and the frequency of the sound. This was followed by the calculation of five parameters: high frequency (Hz), low frequency, bandwidth (Hz), peak frequency (Hz), and call duration (ms). Subsequently, MATLAB R2021a software was utilized to calculate the sound source level (the sound pressure level at a distance of one meter from the sound source) of every call. The formula for source level is as follows:

$$SL=20\log_{10}\left(\frac{P_{1m}}{P_{ref}}\right)$$

Among them, P_1m_ represents the sound pressure value at a distance of 1 m from the sound source (unit: µPa), and P_ref_ represents the reference sound pressure (in air, it is 20 µPa).

Because the advertisement calls of *P. terentievi* had a sentence structure, we picked out complete sentences with obvious structure and high signal-to-noise ratio from recordings for the calculation of sentence duration, number of calls per sentence, call intervals within a sentence, call rate and the percentage of the duration of the first note in the entire sentence.

### 2.3. Statistical Analysis

In order to analyze the vocal diel rhythm of *P. terentievi*, we counted the number of calls per ten minutes for all recordings. Prior to statistical analysis, all data underwent normality distribution testing. For sample sizes exceeding 50, the Kolmogorov–Smirnov normality test is employed; for sample sizes below 50, the Shapiro–Wilk normality test is utilized. The data was considered to be non-normal if *p* < 0.05; otherwise, the data was normally distributed.

Pearson’s correlation coefficient is employed when the data was normally distributed and Spearman’s correlation coefficient is utilized for non-normally distributed data. Given that anura exhibits circadian rhythms, we employed not only the whole-day data but also conducted separate analyses of daytime and nighttime data to examine the correlation between temperature/humidity and the number of calls per 10 min. A correlation analysis between the number of calls per 10 min and temperature and humidity was conducted. The correlation analysis between acoustic parameters and their body size was also performed. We employed the Bonferroni correction to process the results of the correlation analysis between body size and acoustic parameters, which can reduce the probability of false positives. By applying this correction method, we aim to ensure that all detected results remain statistically significant even when multiple comparisons are taken into account [49]. All statistical analyses were performed using SPSS 25.0 software. In order to investigate how temperature and humidity affect the calling rhythm during the day and night, we divided the entire day into two parts: the first half hour before sunrise to the last half hour after sunset constitutes the daytime, and the rest of the time constitutes the night.

The CVs are utilized to estimate the degree of variation in different acoustic parameters among individuals (CV_a_) and within individuals (CV_w_). The formula for calculating CV is as follows:

$$CV=\left(\frac{SD}{X}\right)\times 100\%$$

X denoted the mean of each acoustic parameter, and SD represented the standard deviation [17].

For a kind of acoustic parameter, CV_w_ is calculated as the SD of an individual by its mean value and CV_a_ is calculated as the mean SD of all individuals by their mean value. According to the classification method of CV proposed by Gerhardt [17], CV_w_ was classified into 3 categories: dynamic parameters, static parameters, and intermediate parameters. When CV_w_ is less than 5%, the acoustic parameter is a static parameter; when CV_w_ is greater than 12%, the acoustic parameter is a dynamic parameter; when CV_w_ is between 5% and 12%, the acoustic parameter is an intermediate parameter. In addition, the ratio of CV_a_/CV_w_ is also calculated. When this value is greater than 1.0, it means that the acoustic parameter among individuals is greater than that within individuals, so it can serve as individual identification cues [17].

## 3. Results

### 3.1. The Advertisement Calls of the P. terentievi

A total of 2399 calls from 20 male individuals of *P. terentievi* were identified. These calls were classified into six types through visual and auditory analysis (Table 1). Among them, the A-type advertisement calls had the largest quantity, accounting for 38.2% of the total. The C-type came next, accounting for 31.6% of the total. The E-type had the smallest quantity, accounting for 1.1% of the total.

**Figure 1:**
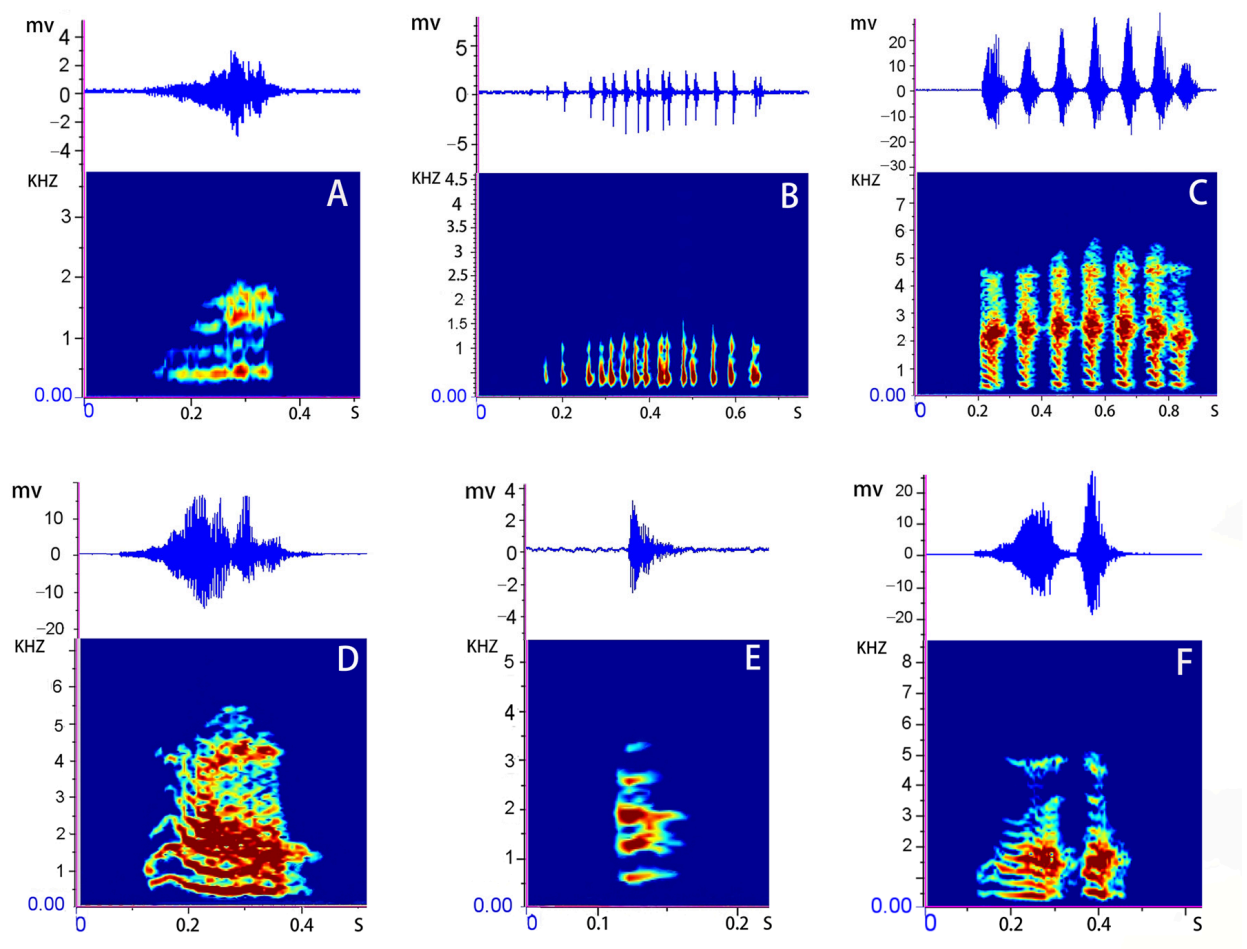
Spectrogram and waveform of six types of advertisement calls for *P. terentievi*. (**A**) A-type call; (**B**) B-type call; (**C**) C-type call; (**D**) D-type call; (**E**) E-type call; (**F**) F-type call. Colors represent the changes in signal energy. The warmer the color, the higher the energy at that frequency at that moment; conversely, the cooler the color, the lower the energy at that frequency at that moment.

The A-type shows an overall “flat sweeping” harmonic. The calls resemble a “woof” and are similar to a dog’s bark. The harmonics range from 5 to 9. The peak frequency is 1119.7 ± 258.4 Hz, the call duration is 121.9 ± 23.2 ms, and the source level is 71.2 dB (Figure 1). This type often appears at the beginning of the vocalization.

The B-type has 6 to 18 pulses and resembles a “gurgling sound”. The peak frequency is 947.5 ± 602.9 Hz, the call duration is 286.1 ± 255.4 ms, and the source level is 59.4 dB. This type always appears at the end of a complete sentence, so we assume that it is an ending word.

The C-type is generally composed of three to nine short notes and every note exhibits a “downward sweeping” harmonic. This type of call sounds like the “quack quack quack” of ducks. The number of harmonics ranges from 8 to 14. The peak frequency is 1981.1 ± 344.5 Hz, the call duration is 330.9 ± 121.9 ms, and the source level is 76.2 dB. During field observations, this type occurs when the frog is at its most excited during each of its vocalizations.

The D-Type is a monosyllabic call with 5 to 18 harmonics, where the harmonics first rise and then fall. This call always sounds like “Ahhhhhhh”. The peak frequency is 1378.1 ± 344.5 Hz, the call duration is 150.9 ± 34.8 ms, and the source level is 75.6 dB.

The E-type is a brief call composed of 5 to 12 harmonics. It sounds like a single short “beep” and always occurs independently during intervals between singing sentences. The peak frequency is 1248.9 ± 1012.1 Hz, the call duration is 29.0 ± 5.8 ms, and the source level is 67.2 dB.

The F-type consists of two notes. The first note exhibits “downward sweep” harmonics and the other shows “upward sweep”. The number of harmonics ranges from 5 to 14. The duration of the first note is generally longer than that of the second one. The peak frequency is 1464.3 ± 258.4 Hz, the call duration is 162.5 ± 34.9 ms, and the source level is 82.3 dB.

### 3.2. Sentence Structure of Advertisement Calls

**Figure 2:**
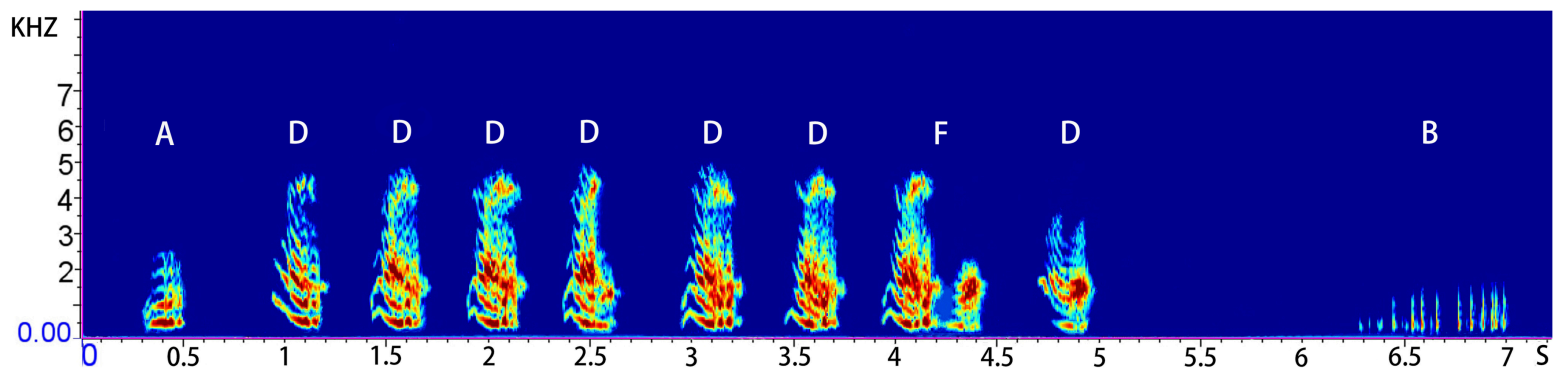
Example 1 for the sentence of *P. terentievi* advertisement calls (the letters represent the types of advertisement calls in this sentence). Colors represent the changes in signal energy. The warmer the color, the higher the energy at that frequency at that moment; conversely, the cooler the color, the lower the energy at that frequency at that moment.

**Figure 3:**
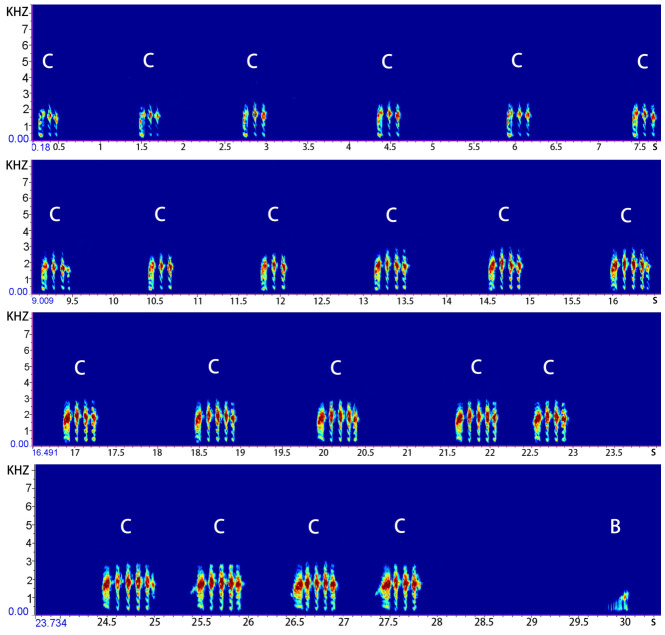
Example 2 for the sentence of *P. terentievi* advertisement calls (the letters represent the types of advertisement calls in this sentence). Colors represent the changes in signal energy. The warmer the color, the higher the energy at that frequency at that moment; conversely, the cooler the color, the lower the energy at that frequency at that moment.

The advertisement calls emitted by *P. terentievi* exhibited a distinct sentence structure. At the beginning of a sentence, the first call often shows the lowest energy compared with the following calls. Subsequent calls typically consist of multiple calls of the same type, arranged at relatively regular intervals. Finally, B-type calls always appear at the end of a complete sentence (Figure 2 and Figure 3). However, their advertisement calls are not always phrased as sentences; sometimes they just emit one of the types of call continuously. The descriptive statistics of the sentence structure were shown in Table 2. The sentence parameters all belonged to dynamic parameters because their CVs exceeded 5% (Table 2).

### 3.3. Coefficients of Variation in Acoustic Parameters

For nearly all acoustic parameters across every call type, both CV_a_ and CV_w_ exceeded 5%, indicating that each acoustic parameter of *P. terentievi* belonged to dynamic parameters. Furthermore, the CV_a_/CV_w_ ratio was greater than 1 for most call types. Only the peak frequency and high frequency of the B-type, as well as the high frequency and bandwidth of the F-type, presented values below 1 (Table 3).

### 3.4. Diel Rhythms of Advertisement Calls

**Figure 4:**
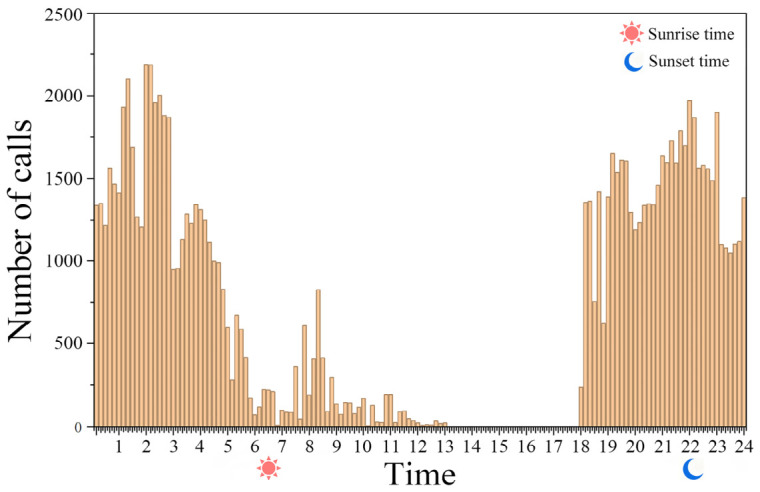
Diel rhythm of the advertisement calls of *P. terentievi* for one day. Each column represents the number of advertisement calls per ten minutes, so the number of columns is 144.

The 24 h calling diel rhythm of *P. terentievi* generally showed that it was most active from after sunset to before sunrise. The advertisement calls began four hours before sunset and then rapidly reached their peak and continued until two hours before sunrise (18:00–the next day 04:30). Subsequently, the calling behavior gradually decreased (04:30–13:00) and ceased after noon until sunset (13:00–18:00) (Figure 4).

### 3.5. Correlation Analysis Between the Number of Calls and Temperature and Humidity

**Figure 5:**
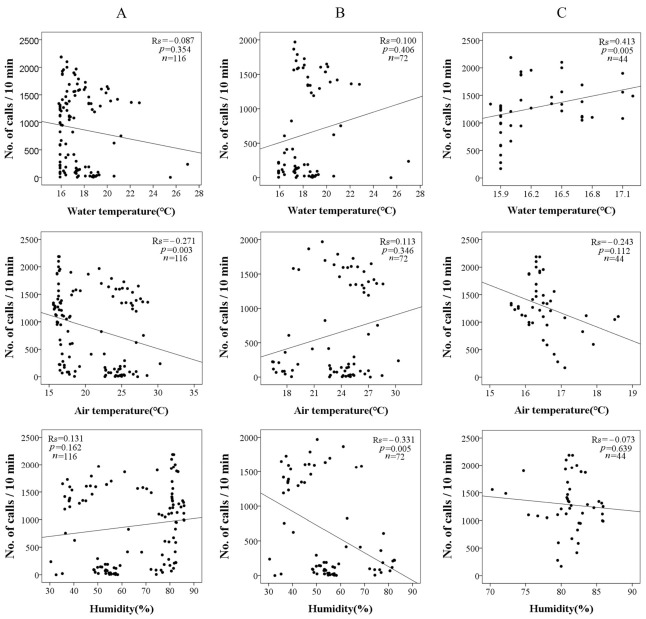
Correlations between calling diel rhythm and temperature and humidity. From left to right: column **A**, over the whole day; column **B**: in daytime; column **C**: at night. The lines in the figure are merely used as visual trend references (based on the Least Squares Method) to visually illustrate the approximate rate of data changes; all statistical significance and correlation strength are strictly based on the non-parametric Spearman *p* test. “Rs” represents the Spearman correlation coefficient, “*p*” indicates the significance of the correlation, and “*n*” denotes the sample size.

Since these data did not conform to the normal distribution (*p* < 0.05) (Table S1), Spearman’s correlation analysis showed that there was a significant negative correlation between the number of calls per 10 min and air temperature (Rs = −0.271, *p* = 0.003) over the whole day. In daytime, the number of calls per 10 min has a significant negative correlation with humidity (Rs = −0.331, *p* = 0.005); at night, the number of calls per 10 min has a significant positive correlation with water temperature (Rs = 0.413, *p* = 0.005) (Figure 5 and Table S2).

### 3.6. Correlation Analysis Between Acoustic Parameters and Body Size

Since the data of body size parameters and acoustic parameters did not conform to the normal distribution (*p* < 0.05) (Tables S1 and S3), we conducted a Spearman correlation analysis and performed Bonferroni correction on the results (Table 4 and Table S4). A-type, B-type, E-type and F-type had no significant correlation between acoustic parameters and body size, while only the calls of C-type and D-type showed a significant correlation with frogs’ body size.

#### 3.6.1. Correlation Analysis Between Acoustic Parameters of C-Type and Body Size

**Figure 6:**
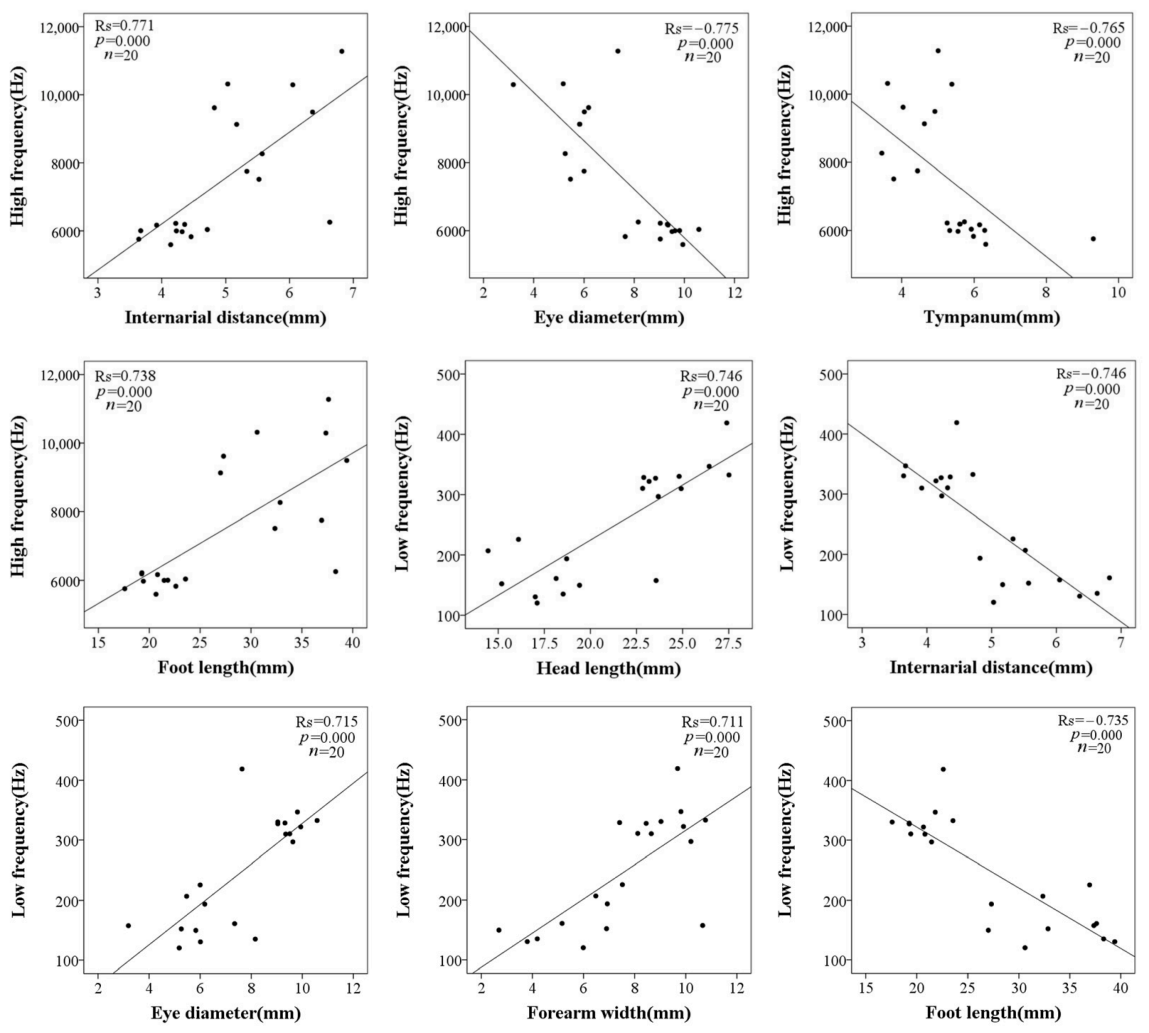
Correlations between acoustic parameters of C-type call and body size. The lines in the figure are merely used as visual trend references (based on the Least Squares Method) to visually illustrate the approximate rate of data changes; all statistical significance and correlation strength are strictly based on the non-parametric Spearman’s *p* test. “Rs” represents the Spearman correlation coefficient, “*p*” indicates the significance of the correlation, and “*n*” denotes the sample size.

The results indicate that the high frequency exhibits a significant positive correlation with the internarial distance and foot length, and a significant negative correlation with the eye diameter and tympanum diameter. The low frequency shows a significant positive correlation with the head length, eye diameter, and forearm width, and a significant negative correlation with the internarial distance and foot length (Table 5 and Table S4, Figure 6).

#### 3.6.2. Correlation Analysis Between Acoustic Parameters of D-Type and Body Size

**Figure 7:**
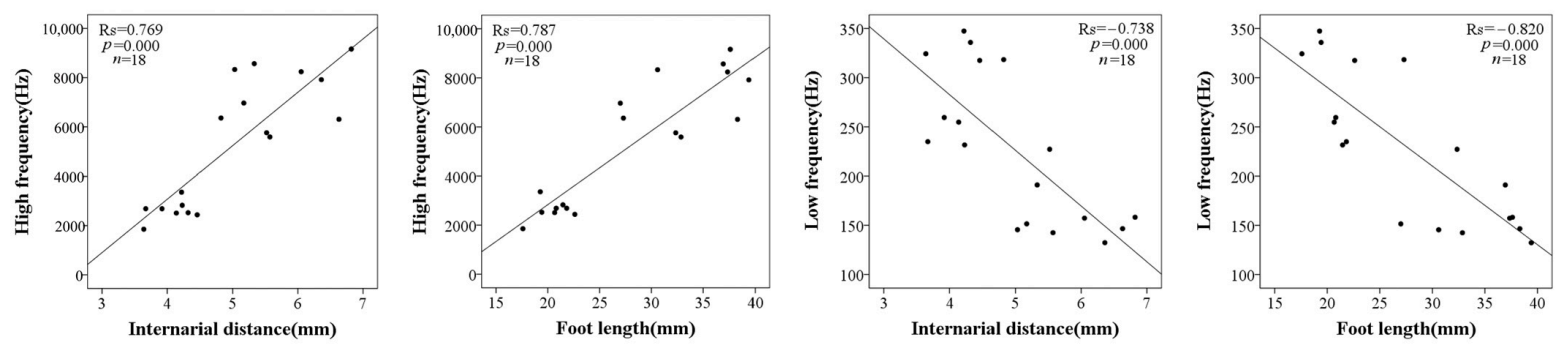
Correlations between acoustic parameters of D-type call and body size. The lines in the figure are merely used as visual trend references (based on the Least Squares Method) to visually illustrate the approximate rate of data changes; all statistical significance and correlation strength are strictly based on the non-parametric Spearman’s *p* test. “Rs” represents the Spearman correlation coefficient, “*p*” indicates the significance of the correlation, and “*n*” denotes the sample size.

The high frequency shows a significant positive correlation with internarial distance and foot length. The low frequency shows a significant negative correlation with the internarial distance with internarial distance and foot length (detailed data are shown in Table 6 and Table S4, Figure 7).

## 4. Discussion

### 4.1. The Diverse Call Types Suggest Complex Communication Functions

Calling is one of the important ways of acoustic communication among individuals of anurans [50,51]. The advertisement calls of frogs play significant roles in courtship, defense and other processes [52,53]. There are six types of advertisement calls of *P. terentievi*, which may imply that the advertisement calls of *P. terentievi* have multiple ecological functions. Vocal animals can achieve territorial defense and convey information about superiority through different call types [54]. The characteristics of calls are the core of intraspecific communication and reproductive behavior [55]. The advertisement call compositions of *P. terentievi* also present diverse patterns, with different advertisement calls composed of different types of advertisement calls in different orders and repetition rates. This implies that *P. terentievi* may convey diverse information through calls. Their call types are almost entirely dynamic parameters and the acoustic parameters of most call types exhibit CV_a_/CV_w_ > 1, indicating that the advertisement calls of *P. terentievi* might possess the function of individual recognition. It is likely that these frogs convey rich individual information through the differences among these parameters [17]. This is similar to the research on *Rana clamitans* [56]. Therefore, the diverse advertisement call types, complex sentence compositions, and high CV_a_ of *P. terentievi* all suggest that its advertisement calls may have complex communication functions.

The advertisement calls of *P. bedriagae* and *P. caralitanus* consist of eight to nine pulses [23,24], while those of *P. ridibundus* are composed of seven to eight short harmonics [26,27,28], and that of *P. nigromaculatus* consists of four to six short harmonics [25]. The duration of each call of the former three species ranges from 590 to 720 ms, and the peak frequency is around 2000 Hz. The call duration of the latter species is 370 to 510 ms, and the average peak frequency is 1200 Hz. The advertisement calls of the above-mentioned species each have only one type. However, there are six types of advertisement calls for *P. terentievi* in Xinjiang, including single-syllable harmonics, multi-syllable harmonics and multi-syllable pulses, and they often appear in the form of sentences. Their average peak frequency is 1681 Hz and call duration varies from 29 to 330 ms. The advertisement calls of *P. terentievi* differ from those of other *Pelophylax* species. We speculate that this might be related to the differences in their habitats. Most species of *Pelophylax* are of plain-dwelling type, while *P. terentievi* is a typical mountain species, only distributed in the waters of mountainous areas or foothills, and has never been observed in plains. However, this requires more extensive ecological research to prove.

### 4.2. The Calling Diel Rhythm Influenced by the Micro-Environment of the Habitat

Numerous studies have found that different anurans exhibit various calling diel rhythms based on their habitats. For example, the calling of *Rhacophorus rhodopus* starts at 19:00 every night and ends at 3:00 the next day [57]; *Crossodactylus gaudichaudii* calls during the day [34]; *Rhacophorus dennysi* calls at night, with the peak calling period occurring in the early hours of midnight [42]; and *Hylarana guentheri* has its peak calling period from 13:00 to 14:00 [55]. Calling behaviors of anurans may be influenced not only by biological factors but also by environmental temperature [35,55]. As a heterothermic animal, their thermoregulatory capacity is extremely limited [3,58]. *P. terentievi* keeps continuously calling from 18:00 (local time) before sunset until 13:00 (local time) before noon the next day. However, they hardly call during the hot noon and afternoon. The correlation analysis of rhythm and temperature/humidity can explain this phenomenon (Figure 5). Therefore, *P. terentievi* prefers to call at times when the water temperature and humidity are relatively high, but the air temperature is not too high. As a mountain species, *P. terentievi* inhabits the bottom of valleys—due to the mountain’s obstruction, sunlight cannot reach the valley bottom after sunrise, keeping its habitat cool (our temperature monitoring data also confirm this point), which is still suitable for its calling, while at around noon, sunlight reach the valley bottom, and the high radiation and high transpiration in arid areas cause the temperature to rise and humidity decrease rapidly [59]. Thus, *P. terentievi* stops calling to protect itself from water loss and heatstroke.

### 4.3. Advertisement Calls May Convey Physical Information

Through the correlation analysis, we found that the advertisement calls of *P. terentievi* may convey information related to head parameters and leg parameters. Most anurans exhibit a negative correlation between their vocal frequency and body size [60] (pp. 637–677). A negative correlation has been found between tympanum diameter and the dominant frequency based on a study of 735 frog species, indicating that larger head structures support lower vocalization frequencies [61]. *Phrynoglossus martensii* shows a negative correlation between head width and the low frequency, meaning that individuals with wider heads emit calls with lower frequency [62]. As a resonant cavity, the larger the head, the lower the resonant frequency [63]. Furthermore, a larger head might indicate a greater hunting ability [64]. And the leg parameters represent the movement ability of the male frog [65]. A greater body weight is an indication of a higher overall survival ability [66]. Typically, female frogs tend to prefer males with low-frequency calls [67,68].

However, our analysis only revealed that two call types are influenced by body size, C and D. On field observation, they are the two types of calls that frogs emit the most when they are at their most excited singing state. *P. terentievi*, with a larger internarial distance and foot length, tends to have higher high frequency and lower low frequency. This indicates that *P. terentievi* individuals with a wider internarial distance and greater mobility can produce higher high frequency to highlight their existence, while also making lower low frequency to showcase their advantageous information for mating. The advertisement calls of *P. terentievi* have a very high sound pressure level and are almost all sung in chorus. In such a noisy environment, to stand out and make one’s call heard, it is the best choice to increase the frequency parameters of the call, because high-frequency signals are more penetrable in the background noise. Whether C-type and D-type calls can convey the male’s advantage information needs to be verified through future behavioral experiments.

## 5. Conclusions

*P. terentievi* has diverse advertisement call types and possesses a variety of sentence structures. Thus, the advertisement calls of this species may carry rich individual information and have complex communication functions. The species has a certain calling diel rhythm which is affected by humidity during the day and by water temperature at night. Furthermore, their acoustic parameters are significantly correlated with their head size and leg size. Males with larger heads and longer legs often make calls at higher frequencies, possibly to stand out in the louder chorus to enhance the efficiency of attracting females. The influence of genetic specificity on bioacoustic parameters in *Pelophylax* frogs is poorly studied, though research reveals complex taxonomy with cryptic forms distinguishable by traits like ‘interval between pulse peaks’ [69]. The study provides basic data for understanding the ecological adaptation mechanism of the acoustic communication strategies of anurans. However, the ecological implications of the complex advertisement calls of *P. terentievi* still need to be revealed and verified through behavioral experiments.

## Figures and Tables

**Table 1:** Descriptive statistics of the advertisement calls of *P. terentievi*.

Call Types	Peak Frequency (Hz)	High Frequency (Hz)	Low Frequency (Hz)	Call Duration (ms)	Bandwidth (Hz)	Signal Types	Number of Notes	Number of Harmonics	Number of Calls	Source Level (dB)
A	1119.7 ± 258.4	4653.1 ± 2284.9	159.2 ± 41.9	121.9 ± 23.2	1033.6 ± 172.3	Harmonic	1	5~9	916	71.2
B	947.5 ± 602.9	2389.4 ± 482.1	144.1 ± 44.4	286.1 ± 255.4	861.3 ± 215.3	Pulse	6–18	-	45	59.4
C	1981.1 ± 344.5	6647.4 ± 4621.9	208.8 ± 144.7	330.9 ± 121.9	1464.3 ± 1119.7	Harmonic	3~9	8~14	758	76.2
D	1378.1 ± 344.5	5820.7 ± 3594.2	162.1 ± 81.1	150.9 ± 34.8	1119.7 ± 538.3	Harmonic	1	5~18	578	75.6
E	1248.9 ± 1012.1	4927.0 ± 2647.7	278.2 ± 95.4	29.0 ± 5.8	1464.3 ± 387.6	Harmonic	1	5~12	26	67.2
F	1464.3 ± 258.4	5958.4 ± 4377.6	201.9 ± 123.4	162.5 ± 34.9	1033.6 ± 409.1	Harmonic	2	5~14	76	82.3

Note: The data presented here are expressed as the median ± interquartile range because they are not normally distributed.

**Table 2:** Descriptive statistics of the sentence structure of *P. terentievi* and their CV_a_.

	Sentence Duration(s)	Number of Calls per Sentence	Call Intervals Within a Sentence(s)	Call Rate	The Percentage of the Duration of the First Note in the Entire Sentence	*n*
Median ± IQR	15.96 ± 14.16	15.55 ± 8.94	0.84 ± 0.59	1.20 ± 0.45	3.21 ± 2.15%	20
CV_a_	88.72%	57.46%	70.11%	37.37%	66.98%	

Note: IQR is the interquartile range for each column of data.

**Table 3:** The CV for *P. terentievi* advertisement calls.

	Peak Frequency			High Frequency			Low Frequency			Call Duration			Bandwidth		
	CV_w_ (%)	CV_a_ (%)	CV_a_/CV_w_	CV_w_ (%)	CV_a_ (%)	CV_a_/CV_w_	CV_w_ (%)	CV_a_ (%)	CV_a_/CV_w_	CV_w_ (%)	CV_a_ (%)	CV_a_/CV_w_	CV_w_ (%)	CV_a_ (%)	CV_a_/CV_w_
A	10.56	29.61	2.80	11.17	19.51	1.75	8.42	21.06	2.50	7.05	13.84	1.96	13.35	19.56	1.47
B	26.31	17.84	0.68	18.58	15.02	0.81	8.88	18.40	2.07	20.37	22.56	1.11	12.89	17.15	1.33
C	3.49	11.30	3.24	13.40	18.10	1.35	17.04	22.30	1.31	7.23	22.40	3.10	26.43	41.10	1.56
D	5.87	17.30	2.95	18.49	17.20	0.93	5.93	21.80	3.68	6.23	16.20	2.60	18.95	24.60	1.30
E	18.32	40.41	2.21	15.10	38.61	2.56	7.20	21.51	2.99	17.52	20.00	1.14	20.19	24.52	1.21
F	7.57	12.70	1.68	11.6	11.50	0.99	8.97	20.80	2.32	15.82	18.10	1.14	22.55	21.00	0.93

**Table 4:** Descriptive statistics of the body size of the *P. terentievi*.

Body Size Parameters	*n*	Minimum	Maximum	Mean ± SD
Snout–vent length (mm)	20	41.80	82.54	70.99 ± 10.43
Head length (mm)	20	14.45	27.51	21.27 ± 4.15
Head width (mm)	20	20.52	33.28	26.41 ± 3.52
Snout length (mm)	20	3.27	12.22	9.80 ± 2.23
Internarial distance (mm)	20	3.64	6.82	4.95 ± 0.96
Interorbital distance (mm)	20	2.40	7.94	3.46 ± 1.18
Width of upper eyelid (mm)	20	3.94	6.29	5.04 ± 0.63
Eye diameter (mm)	20	3.19	10.58	7.62 ± 2.09
Tympanum (mm)	20	3.45	9.30	5.34 ± 1.29
Forearm and hand length (mm)	20	22.54	36.47	29.39 ± 3.70
Forearm width (mm)	20	2.69	10.77	7.62 ± 2.36
Total length of the hindlimb (mm)	20	88.78	137.62	112.54 ± 15.02
Tibia length (mm)	20	23.35	42.68	34.47 ± 5.62
Foot length (mm)	20	17.59	39.41	27.31 ± 7.64
Body weight (g)	20	18.39	54.58	37.89 ± 12.77

**Table 5:** Correlation analysis between C-type acoustic parameters and body size.

	Snout–Vent Length (*n* = 20)	Head Length (*n* = 20)	Head Width (*n* = 20)	Snout Length (*n* = 20)	Internarial Distance (*n* = 20)	Interorbital Distance (*n* = 20)	Width of Upper Eyelid (*n* = 20)	Eye Diameter (*n* = 20)	Tympanum (*n* = 20)	Forearm and Hand Length (*n* = 20)	Forearm Width (*n* = 20)	Total Length of Hindlimb (*n* = 20)	Tibia Length (*n* = 20)	Foot Length (*n* = 20)	Body Weight (*n* = 20)	Low Frequency (*n* = 20)	High Frequency (*n* = 20)	Bandwidth (*n* = 20)	Call Duration (*n* = 20)	Peak Frequency (*n* = 20)
Snout–vent length (mm)		0.087	0.000	0.027	0.701	0.004	0.003	0.089	0.015	0.007	0.339	0.000	0.000	0.582	0.000	0.235	0.310	0.544	0.940	0.650
Head length (mm)	0.392		0.003	0.014	0.001	0.024	0.014	0.001	0.000	0.000	0.000	0.000	0.005	0.006	0.034	0.000	0.003	0.945	0.686	0.042
Head width (mm)	0.782	0.623		0.003	0.663	0.006	0.003	0.013	0.002	0.000	0.056	0.000	0.000	0.840	0.000	0.043	0.095	0.990	0.806	0.627
Snout length (mm)	0.495	0.541	0.623		0.286	0.006	0.018	0.056	0.002	0.000	0.009	0.003	0.005	0.627	0.001	0.243	0.084	0.826	0.591	0.840
Internarial distance (mm)	0.092	−0.681	−0.104	−0.251		0.962	0.165	0.001	0.003	0.156	0.005	0.246	0.384	0.000	0.640	0.000	0.000	0.380	0.705	0.116
Interorbital distance (mm)	0.608	0.502	0.587	0.588	−0.011		0.040	0.296	0.021	0.057	0.133	0.006	0.019	0.995	0.000	0.183	0.616	0.494	0.158	0.441
Width of upper eyelid (mm)	0.628	0.542	0.621	0.522	−0.323	0.462		0.001	0.000	0.025	0.097	0.000	0.000	0.212	0.001	0.066	0.023	0.324	0.910	0.830
Eye diameter (mm)	0.390	0.667	0.542	0.433	−0.686	0.246	0.685		0.000	0.009	0.011	0.001	0.001	0.003	0.074	0.000	0.000	0.127	0.715	0.278
Tympanum (mm)	0.534	0.803	0.656	0.642	−0.630	0.511	0.750	0.750		0.000	0.004	0.000	0.000	0.012	0.007	0.001	0.000	0.552	0.840	0.502
Forearm and hand length (mm)	0.582	0.738	0.844	0.735	−0.329	0.432	0.500	0.569	0.716		0.000	0.000	0.000	0.427	0.001	0.007	0.013	0.280	0.462	0.531
Forearm width (mm)	0.226	0.780	0.433	0.570	−0.603	0.347	0.381	0.557	0.608	0.743		0.006	0.036	0.027	0.173	0.000	0.004	0.980	0.710	0.301
Total length of hindlimb (mm)	0.732	0.723	0.851	0.627	−0.272	0.592	0.734	0.672	0.803	0.844	0.592		0.000	0.582	0.000	0.015	0.019	0.965	0.552	0.462
Tibia length (mm)	0.809	0.603	0.850	0.606	−0.206	0.517	0.755	0.701	0.743	0.786	0.472	0.961		0.734	0.000	0.032	0.016	0.705	0.402	0.498
Foot length (mm)	0.131	−0.594	−0.048	−0.116	0.913	0.002	−0.292	−0.636	−0.549	−0.188	−0.493	−0.131	−0.081		0.373	0.000	0.000	0.175	0.514	0.369
Body weight (g)	0.833	0.477	0.850	0.680	0.111	0.709	0.664	0.408	0.579	0.705	0.317	0.863	0.880	0.211		0.439	0.478	0.960	0.682	0.830
Low frequency (Hz)	0.278	0.746	0.457	0.274	−0.746	0.311	0.418	0.715	0.698	0.582	0.711	0.535	0.481	−0.735	0.183		0.000	0.527	0.565	0.048
High frequency (Hz)	−0.239	−0.629	−0.383	−0.395	0.771	−0.120	−0.506	−0.775	−0.765	−0.544	−0.612	−0.520	−0.532	0.738	−0.168	−0.788		0.319	0.490	0.301
Bandwidth (Hz)	0.144	0.017	−0.003	−0.053	−0.208	0.162	0.233	0.353	0.141	−0.254	−0.006	−0.011	0.090	−0.316	0.012	0.150	−0.235		0.082	0.835
Call duration (ms)	0.018	0.096	−0.059	−0.128	−0.090	0.328	−0.027	0.087	−0.048	−0.174	0.089	−0.141	−0.198	−0.155	−0.098	0.137	0.164	0.398		0.248
Peak frequency (Hz)	0.108	0.459	0.116	−0.048	−0.362	0.183	0.051	0.255	0.159	0.149	0.244	0.174	0.161	−0.212	0.051	0.447	−0.244	0.050	0.271	

Note: The data in the right upper triangle represent the *p* value after Bonferroni correction, while the data in the left lower triangle represent Spearman’s correlation coefficient (Rs). After Bonferroni correction, α = 0.00026. Therefore, a significant correlation exists only when *p* < 0.00026.

**Table 6:** Correlation analysis between D-type acoustic parameters and body size.

	Snout–Vent Length (*n* = 18)	Head Length (*n* = 18)	Head Width (*n* = 18)	Snout Length (*n* = 18)	Internarial Distance (*n* = 18)	Interobital Distance (*n* = 18)	Width of Upper Eyelid (*n* = 18)	Eye Diameter (*n* = 18)	Tympanum (*n* = 18)	Forearm and Hand Length (*n* = 18)	Forearm Width (*n* = 18)	Total Length of the Hindlimb (*n* = 18)	Tibia Length (*n* = 18)	Foot Length (*n* = 18)	Body Weight (*n* = 18)	Low Frequency (*n* = 18)	High Frequency (*n* = 18)	Bandwidth (*n* = 18)	Call Duration (*n* = 18)	Peak Frequency (*n* = 18)
Snout–vent length (mm)		0.179	0.000	0.054	0.616	0.009	0.007	0.132	0.020	0.017	0.598	0.002	0.000	0.515	0.000	0.926	0.657	0.311	0.317	0.372
Head length (mm)	0.331		0.014	0.059	0.001	0.071	0.023	0.007	0.000	0.002	0.001	0.002	0.025	0.005	0.106	0.009	0.003	0.693	0.782	0.254
Head width (mm)	0.808	0.569		0.004	0.887	0.012	0.005	0.047	0.003	0.000	0.123	0.000	0.000	0.735	0.000	0.971	0.307	0.206	0.195	0.067
Snout length (mm)	0.461	0.453	0.639		0.358	0.023	0.029	0.144	0.003	0.001	0.039	0.016	0.023	0.717	0.006	0.791	0.140	0.717	0.735	0.489
Internarial distance (mm)	0.127	−0.705	−0.036	−0.230		0.852	0.272	0.002	0.011	0.216	0.006	0.295	0.499	0.000	0.458	0.000	0.000	0.735	0.129	0.861
Interorbital distance (mm)	0.599	0.436	0.580	0.534	0.047		0.061	0.512	0.032	0.110	0.336	0.023	0.056	0.760	0.001	0.486	0.711	0.399	0.747	0.049
Width of upper eyelid (mm)	0.613	0.531	0.634	0.514	−0.274	0.450		0.002	0.000	0.036	0.144	0.000	0.000	0.279	0.003	0.362	0.061	0.327	0.368	0.203
Eye diameter (mm)	0.369	0.613	0.473	0.358	−0.685	0.165	0.685		0.000	0.031	0.049	0.004	0.001	0.003	0.175	0.006	0.001	0.048	0.801	0.534
Tympanum (mm)	0.542	0.806	0.651	0.653	−0.585	0.506	0.771	0.758		0.001	0.009	0.000	0.000	0.028	0.012	0.031	0.002	0.494	0.990	0.443
Forearm and hand length (mm)	0.554	0.674	0.837	0.730	−0.307	0.389	0.497	0.510	0.711		0.001	0.000	0.000	0.553	0.002	0.299	0.064	0.874	0.732	0.385
Forearm width (mm)	0.133	0.717	0.377	0.490	−0.620	0.241	0.358	0.470	0.593	0.705		0.027	0.142	0.020	0.428	0.021	0.016	0.893	0.065	0.693
Total length of hindlimb (mm)	0.688	0.684	0.876	0.558	−0.261	0.531	0.739	0.646	0.814	0.833	0.519		0.000	0.587	0.000	0.287	0.062	0.213	0.418	0.168
Tibia length (mm)	0.779	0.527	0.891	0.534	−0.170	0.457	0.764	0.693	0.752	0.763	0.360	0.950		0.766	0.000	0.453	0.073	0.099	0.266	0.165
Foot length (mm)	0.164	−0.633	0.086	−0.092	0.924	0.077	−0.270	−0.650	−0.517	−0.150	−0.542	−0.137	−0.075		0.276	0.000	0.000	0.735	0.168	0.964
Body weight (g)	0.806	0.393	0.913	0.624	0.187	0.707	0.652	0.335	0.579	0.676	0.199	0.822	0.837	0.271		0.699	0.861	0.303	0.103	0.046
Low frequency (Hz)	0.024	0.598	0.009	0.067	−0.738	0.176	0.228	0.623	0.509	0.259	0.538	0.265	0.189	−0.820	−0.098		0.003	0.926	0.017	0.705
High frequency (Hz)	−0.112	−0.653	−0.255	−0.362	0.769	−0.094	−0.449	−0.694	−0.690	−0.445	−0.558	−0.449	−0.432	0.787	−0.044	−0.662		0.458	0.564	0.717
Bandwidth (Hz)	0.253	0.100	0.313	−0.092	−0.086	0.212	0.245	0.472	0.172	0.040	0.034	0.309	0.401	−0.086	0.257	0.024	−0.187		0.070	0.303
Call duration (ms)	−0.250	0.070	−0.320	0.086	−0.372	−0.082	−0.225	0.064	−0.003	0.087	0.443	−0.204	−0.277	−0.340	−0.397	0.556	−0.146	−0.437		0.274
Peak frequency (Hz)	0.224	0.284	0.441	0.174	−0.044	0.470	0.315	0.157	0.193	0.218	0.100	0.340	0.342	−0.011	0.476	0.096	−0.092	0.257	−0.273	

Note: The data in the right upper triangle represent the *p* value after Bonferroni correction, while the data in the left lower triangle represent Spearman’s correlation coefficient (Rs). After Bonferroni correction, α = 0.00026. Therefore, a significant correlation exists only when *p* < 0.00026.

## Data Availability

The original contributions presented in this study are included in the article/Supplementary Materials. Further inquiries can be directed to the corresponding author.

## Supplementary Materials

The following supporting information can be downloaded at: https://www.mdpi.com/article/10.3390/biology15151304/s1, Table S1: Normality test tables for various parameters; Table S2: Correlation analysis between calling diel rhythm and temperature and humidity; Table S3: The original data on the body size and acoustic parameters of the *P. terentievi*; Table S4: Correlation analysis table of acoustic parameters of different advertisement call types and body size.

## Abbreviations

The following abbreviations are used in this manuscript:

CV	Coefficient of variation
CV_a_	Coefficient of variation between individuals
CV_w_	Coefficient of variation within individuals
